# Reducing inequities in maternal and child health in rural Guatemala through the CBIO+ Approach of Curamericas: 7. The empowering effect of Care Groups

**DOI:** 10.1186/s12939-022-01759-5

**Published:** 2023-02-28

**Authors:** Corey Gregg, Mario Valdez, Ira Stollak, Shayanne Martin, William T. Story, Henry B. Perry

**Affiliations:** 1 Louisiana State University Health Sciences Center, New Orleans, Louisiana USA; 2 Curamericas/ Guatemala, Calhuitz, San Sebastián Coatán, Huehuetenango, Guatemala; 3 Curamericas Global, Raleigh, North Carolina USA; 4 Institute for Global Health Sciences, University of California San Francisco, San Francisco, California USA; 5 Department of Community and Behavioral Health, University of Iowa College of Public Health, Iowa City, Iowa USA; 6 Health Systems Program, Department of International Health, Johns Hopkins Bloomberg School of Public Health, Baltimore, Maryland USA

**Keywords:** Women’s empowerment, Social capital, Maternal health, Child health, Community health, Primary health care, Community-based primary health care, Implementation research, Based, Impact-Oriented Approach, Care Groups, Community birthing centers, Guatemala, Equity, Curamericas Global, Curamericas/Guatemala

## Abstract

**Background:**

While there is extensive published evidence regarding the effectiveness of the Care Group Approach in promoting community-wide health behavior change, there is no published evidence regarding its empowering effect on its participants. Our study aimed to understand if the Care Group Approach as applied in the Curamericas/Guatemala Maternal and Child Health Project in isolated rural mountainous communities in Guatemala produced evidence of empowerment among the female participants. This is the seventh of 10 papers describing the expanded Census-Based, Impact-Oriented (CBIO+) Approach in improving the health and well-being of mothers and children in the rural highlands of the Department of Huehuetenango, Guatemala.

**Methods:**

We conducted semi-structured individual and group interviews with 96 female Care Group participants –including Level-1 Care Group Promoters, Care Group Volunteers, and Self-Help Group participants. The participants were from six communities – two from each of the three municipalities making up the Project Area. Data were analyzed both using deductive thematic and by exploring the following social constructs: perceived social status, self-efficacy, decision-making autonomy, and formation of social capital.

**Results:**

The findings supported the hypothesis that Care Group participation was an empowering process. The primary themes that emerged included increased respect accorded to women in the community, women’s willingness and ability to make decisions and their confidence in making those decisions, and the development of stronger bonds among Care Group members, with other community members, and with community leaders.

**Conclusion:**

Through increased theoretical and practical knowledge about important maternal and child health matters and through the social experience of obtaining this knowledge and sharing it with other community members, participation in the Care Group Approach empowered participants to make positive health behavior changes for themselves and for their children and families. This, in turn, led many participants to become more engaged in community activities for improved health and beyond, thereby enhancing social capital in the community. We conclude that the Care Group Approach, as applied in this setting, has made it possible for marginalized indigenous women living in a male-dominated society to become more empowered.

## Background

Between 2011 and 2015, Curamericas/Guatemala implemented a community-based primary health care program in the entire municipalities^1^ of San Sebastián Coatán, San Miguel Acatán, and Santa Eulalia, which have a combined population of 98,000. We refer to this integrated package of services as the Curamericas Maternal and Child Health Project (hereafter referred to as the Project). The goal of the Project was to improve the health of mothers and children through an empowering participatory process. The Project utilized three implementation methodologies: (1) the Census-based, Impact-oriented (CBIO) Approach [1], (2) the Care Group Approach [2], and (3) *Casas Maternas Rurales* (Community Birthing Centers) [3]. These were combined into an integrated package of services in collaboration with the existing health services of the Guatemala Ministry of Public Health and Social Welfare Health (*Ministerio de Salud Pública y Asistencia Social*). We refer to these three implementation methodologies in combination as CBIO+.

This article is part of a series of 10 articles that summarize the findings of an evaluation of the Project [4–12]. In this article we describe changes in aspects of women’s empowerment, including perceived respect/social status, self-efficacy, decision-making autonomy, and formation of social capital, as reported by participants in the Care Group activities of the Project.

The Care Group Approach is a health promotion model based on volunteerism, peer-to-peer education, and outreach to all households. By means of a “cascade” approach, an increased number of trainers are trained by other trainers, making it eventually possible to reach every household with key health and nutrition messages [2, 13–15]. The Care Group Approach has been successfully implemented by various community-based health programs in Africa, South Asia, and Latin America since the 1990s. Of note, the Care Group Approach has reached a large number of individual households and families at a low cost, with notable increases in population coverage of interventions that improve maternal and child health (e.g., adoption of healthy household behaviors, utilization of maternal and child health services, and reductions in the morbidity and mortality of children younger than 5 years of age) [2, 15–17].

There is a strong impression among those with direct experience with the Care Group Approach that participation in Care Groups has also empowered its female participants who often have low levels of education and live in contexts of severe poverty and in households overly dominated by men. However, to date there has been no evidence in the peer-reviewed literature that provides any evidence to support this impression.

### Care Group structure

**Fig. 1:**
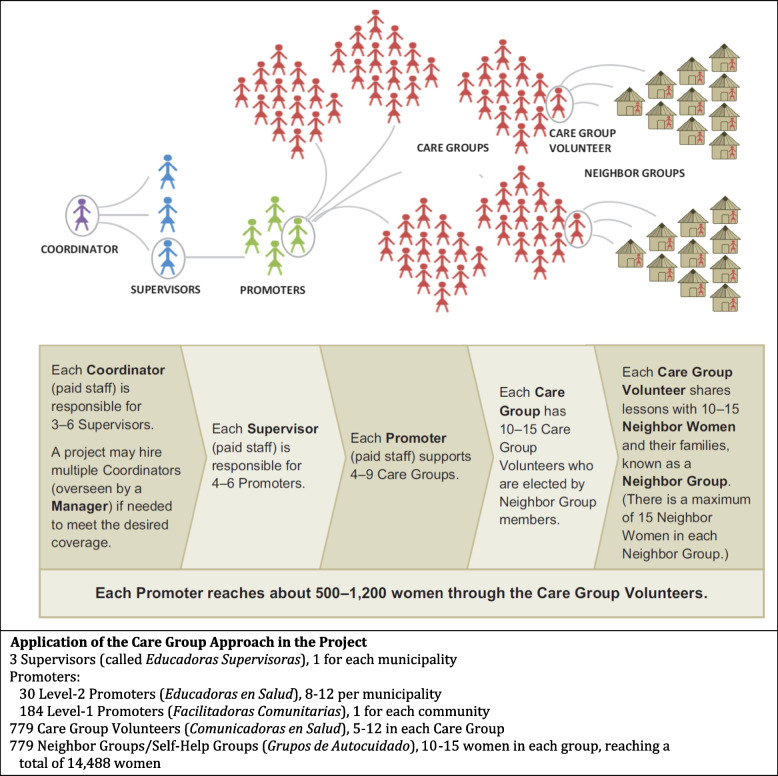
The Care Group training cascade and its application in the Curamericas/ Guatemala Maternal and Child Health Project, 2011–2015﻿. Source: Figure obtained from Perry et al. [2]

The first paper in this series [4] describes the Care Group structure and processes that the Project implemented. The Care Group methodology and its effectiveness more broadly are described elsewhere [2, 18]. Briefly, as implemented in the Project, a Care Group (*Grupo de Cuidado*) is composed of 5–12 women Care Group Volunteers (*Comunicadoras*) who meet together every 2 weeks with a paid Level-1 Promoter (*Facilitadora Comunitaria*) to learn one or a small number of health messages that they each share with their neighbors. Care Group Volunteers are selected by the community to represent 10–15 households with a pregnant woman or mother of a young child which are near their own home in such a way that all households in the community with a child younger than 2 years﻿ of age or a pregnant woman are linked to a Care Group Volunteer*.* Level-1 Promoters functioned as Care Group Promoters and trained the Care Group Volunteers in their village. The Level-1 Promoters were not salaried staff, but rather volunteers receiving a stipend. They were also mothers of under-2 children and among the Project’s beneficiaries. During the subsequent 2 weeks, each of the Care Group Volunteers would meet with the mothers of the assigned households, either as a group (called a Self-Help Group) or individually during a visit to the woman's home to share ﻿and discuss the message(s) she had just learned from her Level-1 Promoter*.* When the Care Group﻿ met again, they would discuss their experiences and learn a new message. They would also report any vital events (births or deaths) that may have occurred among their neighbors, which formed the basis of our mortality assessment (analyzed in Paper 5 in this series [8]. Fig. 1 depicts the Care Group structure. All of the direct participants in the Care Group process (Level-1 Promoters, Care Group Volunteers, and Self-Help Group participants) were women.

### Women’s empowerment

Following Kabeer, we define empowerment as “… the process by which those who have been denied the ability to make [strategic life] choices acquire such an ability” [19]^P. 437^. ﻿Furuta et al. argue that the measurement of women’s empowerment should include resources (including material, human, and social), agency (including decision-making autonomy), and health-related outcomes [20]. Women and children living in the context of severe poverty and gender discrimination often have low rates of health care utilization and poor health outcomes [20–23]. As described below, there is evidence from other rural, low-income settings that gains in social resources and agency related to women’s empowerment – as expressed by improved social status, increased self-efficacy, increased decision-making autonomy, and higher levels of social capital – are associated with improvements in maternal and child health outcomes.

First, one's opinion about her social ranking (i.e., subjective social status) is a robust predictor of morbidity and mortality and is not fully explained by objective measures of socioeconomic status (e.g., education, occupation, and income) [24, 25]. Subjective (i.e., perceived)﻿ social status may influence health in three ways: (1) social structures that influence life opportunities, (2) physiological responses to stress that lead to increased biological vulnerability to disease; and (3) unhealthy behaviors linked to coping mechanisms [26]. In the context of maternal and child health, a woman’s perceived social status in her community is positively associated with her physical and emotional health during pregnancy and the postpartum period [27].

Second, according to Bandura [28], self-efficacy is the belief in one’s ability to control his or her own behavior and is the foundation for motivation and action. Those with high efficacy expect to overcome obstacles and achieve positive outcomes, while those with low efficacy expect their efforts to be insufficient to produce positive outcomes [28]. Improvements in self-efficacy have been shown to improve maternal and child health outcomes by increasing a woman’s confidence in her ability to use maternal and child health services [29].

Third, women’s decision-making autonomy is defined as “the control women have over their own lives—the extent to which they have an equal voice with their husbands in matters affecting themselves and their families” [30]^p. 688^ Prior research has demonstrated that women’s status (specifically, agency and autonomy) is positively correlated with the health of women due to better partner communication and improved access to reproductive health services [31, 32]. Women’s autonomy has demonstrated a persistent positive relationship with maternal health service utilization and pregnancy outcomes by enabling pregnant women to act on their preferences for health care and take control of their own health needs [33–35]. This is especially important in societies where men control most household resources and do not always prioritize women’s health needs [20, 36]. 

Fourth, social capital—bonds of mutual trust and support between community members, which affect the allocation of resources [37]—is associated with lower levels of mortality, better self-rated health, and healthier behaviors [38].

Social capital is typically categorized into two forms—structural and cognitive [39]. Structural social capital primarily reflects Bourdieu’s conceptualization of social capital as resources embedded in social networks [40], whereas cognitive social capital aligns more closely with Putnam’s concepts of social trust, reciprocity, and effective norms [41]. The literature further distinguishes between bonding and bridging social capital, which have structural and cognitive components. Bonding social capital refers to social connections between socially similar individuals, whereas bridging social capital refers to connections between socially dissimilar individuals [42]. In the context of maternal and child health, social capital has been shown to be associated with improvements in health care utilization and health outcomes [43, 44]. This association is likely due to improving women’s access to material and social resources through trusted social relationships [43].

In﻿ societies in which women are marginalized, it is possible that the Care Group Approach empowers women through these four sociological processes, which enable them to improve their own health as well as the health of their families and communities.

### The status of rural indigenous women in Guatemala

Women in Guatemala, especially indigenous rural women, and particularly those in the Project Area, encounter numerous sociocultural barriers to their health and the health of their families. A culture of *machismo* exists throughout Guatemala, but it is prominent in the rural indigenous areas where, in the male-dominated traditional Maya culture, women have limited control over their bodies, limited decision-making autonomy as well as limited social participation in public spaces [45]. After marriage, women commonly live with the husband’s household that often includes her mother-in-law, who exerts considerable control over household nutritional practices [45]. These newly married women have little control over finances or other daily decisions and limited mobility to leave their house without their husband’s consent. Domestic violence in the Project Area is not uncommon. Disempowerment is also reflected in high rates of female illiteracy and, in particular, low levels of health knowledge. This threatens women’s self-confidence and self-efficacy in making health-related decisions as well as contributes to the high level of maternal mortality [3, 46].

### Research questions

This paper focuses specifically on the effect of Care Groups on the women who participate in the Care Group process. Given the gender inequality present in the Project Area and given the strong impression of one of us (HP) on the basis of personal experience in the evaluation of five Care Group projects elsewhere that participation in the Care Group process is empowering and that it creates strong social bonds among Care Group participants, the Project evaluation team decided to assess whether participation in the Care Group Approach led to increased perceived social status (as measured by the increased respect from others received by Care Group participants), enhanced self-efficacy (as measured by increased control over their lives), improved decision-making autonomy (as measured by increased power to make decisions that affect their lives), and increased social capital (as measured by increased social bonds among Care Group participants and with others in the community outside of the Care Group participants). Specifically, the evaluation explored the three following research questions:

1. Perceived social status: In what ways did Level-1 Promoters, Care Group Volunteers, and Self-Help Group members think that their participation in the Care Group Approach helped improve their status in the community?
2. Self-efficacy and decision-making autonomy: Did the Level-1 Promoters, Care Group Volunteers, and Self-Help Group members feel they had more control over their lives and the decisions that affect their lives following their participation in the Care Group Approach?
3. Social capital: Did the Level-1 Promoters, Care Group Volunteers, and Self-Help Group members feel that they could participate more freely in community meetings and community activities following their involvement in the Project?

## Methods

Details about the setting and the overall research methodology are located elsewhere in this series [5]. For the purpose of this research, a deductive thematic analytical approach was used to identify a priori concepts [47]. This approach was used to systematically examine four social constructs associated with women’s empowerment and maternal and child health: perceived social status, self-efficacy, decision-making autonomy, and formation of social capital.

Nine interview questions and 21 follow-up questions were designed to elicit information necessary to answer the three primary research questions mentioned above. These questions are contained in the Appendix. Three interviewers with at least a secondary-level education, native speaking ability of the local Maya language, and fluency in Spanish were hired from each of the three municipalities represented in the study. The nine interviewers were trained in the methods of in-depth and group interviews, as well as in the purpose of the Project, the fundamentals of qualitative evaluation, and the content of the interviews. The interview questions had been previously translated from English to Spanish using a team of two bilingual native English speakers and three Guatemalan native Spanish speakers. The interviewers then collaboratively translated each interview question from Spanish into the local Maya languages used in the Project Area (Chuj, Akateko, Q’anjobal).

The communities included in the study—Ququilum and Jajhuitz in the municipality of San Sebastian Coatán, Paiconop Grande and Aldea Poza in the municipality of San Miguel Acatán, and Altamiranda and Kanajaw Xixilack, in the Santa Eulalia municipality—were chosen purposively by Curamericas/ Guatemala staff. The staff considered that these communities were representative of the Care Group experience in each municipality and they were also readily accessible.^2^ As described elsewhere [4, 5], the Project was implemented in Areas A and B. Area A has ﻿89 communities and a total population of 44,833 people. In Area A, the communities received the CBIO and Care Group elements of the Project for the full four years of the Project beginning in 2011. Area B, with an additional 91 communities having a total population of 45,052 people, received these services for the final 20 months of the Project (October 2013 until May 2015). The Area A communities were scattered throughout each of the three municipalities as were the Area B communities. Participants were selected from one community in each municipality in Area A and from one community in each municipality in Area B (for a total of six communities).

In each selected community, the Level-1 Promoters, the Care Group Volunteers, and members of a randomly selected Self-Help Group were interviewed. Members of Self-Help Groups that were interviewed were randomly sampled to ensure equity in selection. 6–8 Care Group Volunteers in each community were interviewed along with 8–9 Self-Help Group members. A total of 6 Level-1 Promoters, 6 groups of 6–8 Care Group Volunteers, and 6 groups of 8–9 Self-Help Group participants were included in these interviews. The Level-1 Promoters were paid and had at least a secondary education. They supervised the Care Group Volunteers, who were selected by their peers and community leaders. The Self-Help Group members were mothers who were in the catchment area of the Care Group Volunteer.

Data collection methods included key-informant interviews﻿ and group interviews. Interviewers carried out in-depth interviews with Level-1 Promoters and group interviews with Care Group Volunteers and Self-Help Group participants. The group interview approach was used in order to obtain information from as many of the Care Group Volunteers and Self-Help Group participants as possible given time and resource constraints.

The group interviews were not focus group discussions, which are designed to catalyze conversation in response to a lead question. Instead, the group interviews consisted of natural groups of individuals embedded in the Project that typically know each other. Natural groups are a common method used in community-based research and are more informal than focus groups. Natural groups are often used to due to time limitations for data collection and analysis, which was the case in this study [48]. These group interviews followed a structured interview guide by asking the group members to respond individually to a question, and then moving to the next question.

Interviews were conducted in the local Maya language of each municipality during the Project’s final evaluation in May 2015. As one interviewer asked questions, another wrote down verbatim or paraphrased responses in Spanish (translated in real time), and a third noted behaviors in the group. The team reviewed the written transcripts immediately after the interviews and, based on their recall of the responses, verified the accuracy of the translations written in the transcript and made corrections when necessary. Since the interviews were not recorded, this process ensure the accuracy of the transcripts.

The notes in Spanish from the in-depth and group interviews were translated into English for evaluation and analysis by a bilingual Project evaluator. We analyzed the interview data using deductive thematic analysis focused on four social constructs: perceived social status, self-efficacy, decision-making autonomy, and formation of social capital. Following translation to English, the data were analyzed for themes using a combination of open and axial coding [49], which gave rise to four distinct themes, each possessing several sub-themes. A deductive assessment was carried out regarding the social constructs mentioned above. Here we describe the results of this study, deconstructing these social constructs that were uncovered from the exploration of women’s empowerment: increased self-respect/social status among Care Group participants; increased self-efficacy and decision-making autonomy; and increased social capital for the participants within their own social networks. We refer to increased social capital within Self-Help Groups as bonding social capital while we refer to increased social capital for the participants with other community groups and with community leaders as bridging social capital.

## Results

The respondents described many concrete examples of four areas of empowerment that their participation in Care Groups produced. Study participants stated that community members were asking them more frequently for advice and opinions (both related and unrelated to Care Group topics), indicating an increase in their social status. Study participants asserted that they had more confidence in their own decisions due to knowledge gained through their Care Group participation, providing evidence of increased self-efficacy. Study participants reported that they were currently making more of their own decisions compared to before their participation in Care Groups, indicating an expansion of their decision-making autonomy. Finally, study participants said that their participation in the Care Group experience led to considerably stronger friendships among those in the Care Groups and in the Self-Help Groups as well as to new relationships and collaborations with others in the community with whom they previously had limited interactions, most notably with community leaders. This is evidence of increased social capital formation. Specific details of these findings are described below.

### Increased self-respect/social status

A frequently described benefit of the Care Group Approach was an increase in social status achieved through participation. The Level-1 Promoters reported increased status in their communities because of their leadership role and knowledge gained through their participation. Notably, the Care Group Volunteers and the Self-Help Group participants described increased social status through their roles as participants.

*Because we have participated in a lot of trainings … they* [our neighbors] *ask us for our opinions.*

*-*﻿Care Group Volunteer

Some Care Group Volunteers reported that community members started to ask for their advice at community meetings as trusted, respected women. Likewise, Self-Help Group participants reported an increase in their social status since they began to be viewed as trusted members of the community and to be called upon for help during medical illnesses and emergencies.

*The people come to us for help because of what we have learned.*

*-*Self-Help Group participant

Care Group Volunteers reported that they were participating more frequently in the community and were asked for their opinions by community leaders. Likewise, some Self-Help Group participants also spoke of an increased ease of participation in community activities, which they ascribed to the knowledge gained in the Self-Help Groups that led the community to view them as having greater worthiness and value to the community. Several women stated they were initially timid about speaking in public, but the experience gained through the Care Groups rid them of this fear. Less timidity resulted in increased participation in community meetings and activities. They also expressed the importance of gaining experience in speaking their minds at the Care Group and Self-Help Group meetings, which they believed to have helped them speak confidently in public situations other than in the Care Groups. They stated that this confidence emboldened them to participate at community meetings.

*We believe that we can participate more easily in community activities and meetings because now we have lost our fear as a result of the trainings that have been given to us. Now we have knowledge.*

*-*Care Group Volunteer

*We can now participate easily* [in community meetings] *because, through the trainings, we obtained knowledge about our rights to participate in community activities.*

*-*Self-Help Group participant

### Increased self-efficacy and decision-making autonomy

Many participants also reported increased confidence in their own decisions as well as an improved ability to implement those decisions in their own households and in the community. One Level-1 Promoter indicated she gained greater power over her own body from her new knowledge of and access to family planning.

*The Educadora* [Level-2 Promoter] *taught me how to care of myself and when to seek assistance from a doctor. Now, I can make my own decisions and to have children if I want to.*

*-*Level-1 Promoter

The Care Group Volunteers related that, since the beginning of the Project, their confidence in their own abilities had increased, leading them to make more of their own decisions. Furthermore, they stated that they were now able to make those decisions due to their diminished fear of developing new social relationships and engagement with the community, as well as due to their new awareness of their own value acquired through the Care Group experiences. They reported they no longer feared their mothers-in-law, public speaking, or public action. One *Comunicadora* (Care Group Volunteer) reported that her role made it easier for her to ask community leaders about new community activities that may benefit her and her family.

*As women lost their fear, now they have seen our participation in various activities that are happening in our communities.*

*-*Care Group Volunteer

Care Group Volunteers attributed their increased confidence in decision making and increased autonomy in their personal lives to the knowledge gained from the Project. Specifically, from what they learned through the Care Group sessions, the Care Group Volunteers and the Self-Help Group participants described comfort in making decisions regarding when to seek medical assistance during pregnancy, how to care for a sick child, how to feed their family appropriately, and how to improve the sanitation and hygiene in their homes. Additionally, some Care Group Volunteers indicated that they had adopted family planning, resulting in greater control over their own bodies and finances. Many of them also indicated that they had increased control over their lives as a result of the knowledge gained through the Care Group experience.

*The truth is that we now have more knowledge and know the importance of the* [family planning] *topics, and there are situations in which we find ourselves now being able to make our own decisions.*

*-*Care Group Volunteer

*Yes* [we have more control over our lives] *because, before we were Comunicadoras* [Care Group Volunteers], *we would do what our elders told us to do, such as “don’t give colostrum”* [to our newborns]. *Sometimes our mothers-in-law were in charge of caring for our newborns and they would give them coffee or sugary drinks, but now we do not let them do this thanks to the teachings that we were given. Today our young mothers take good care of their children, and they also have knowledge about family planning.*

*-*Care Group Volunteer

*We can decide how many children we want to have, so that we can support them well.*

*-*Care Group Volunteer

Like Level-1 Promoters and Care Group Volunteers, many Self-Help Group participants described increased decision-making autonomy and decreased domination by their male partners. According to the women, the knowledge they gained enabled them to make decisions in their households as well as in the community. Additionally, this newfound knowledge led to greater confidence in making personal decisions.

*Yes, we have more* [confidence] *because now we are informed, and we are capable of deciding for ourselves*

-Self-Help Group participant

In one interview, Self-Help Group participants noted they had gained confidence in their ability to make decisions as a result of practicing the skill of decision-making during their group sessions. The women stated that they now take ownership of their health and the health of their family. They described new experiences of standing up to mothers-in-law and husbands, and nearly every woman interviewed stated she now had more control over her own life. They also reported losing fear of participating in community meetings.

[We have] *more control because now we can decide things without consulting anybody, and now we realize the importance of our own opinions.*

-Self-Help Group participant

*Yes, we can participate more easily because now we have lost the fear and the shame of speaking in the community meetings and activities. For example, now we only notify our husbands that we are going to a community meeting or activity* [instead of having to ask for permission]*.*

-Self-Help Group participant

*Before, we participated very little. Now, we like to participate in any meeting that there might be, health-related or otherwise. We participate and they value our opinions.*

-Self-Help Group participant

Regarding the source of their newly discovered confidence, Self-Help Group participants viewed the Project positively because they observed improved health outcomes, and many expressed a desire for the Care Group activities to continue. They believed their children to be sick less often, and they believed the knowledge gained through the Project was inherently valuable—for their own health and for the health of their children. According to one Self-Help Group participant:

*We like it because they* [the Care Group Volunteers] *train us to care for our children because now they tell us which foods are good for them and also which are bad, which foods we can feed them and which ones we should not feed them, and also how we can teach them to wash their hands. Before, I would give chicharrones* [deep fried pork rinds] *to my children, but this is bad, and it is better to give them fruits. We like that about our group*.

-Self-Help Group participant

Care Group Volunteers, most of whom had very little formal education, emphasized the importance of understanding the causes of illness and the benefits of healthy living as reasons for their behavior change. An understanding of the reasons for behavior change—as opposed to simply following the directions of the teachers—served to increase confidence in their own decisions.

*Regarding changes that I have seen in my own life; before I did not give the most nutritious foods to my children. Now I do because it is beneficial to them and to me.*

-Care Group Volunteer

*Before we would not do what we practiced* [during the Care Group meetings]*. Today, we have changed. For example, as mothers, we now know the importance of colostrum, that it serves as the baby’s first vaccine, and also* [the importance of] *hygiene* [handwashing] *before eating.*

*-*Care Group Volunteer

Self-Help Group participants understood that certain health behaviors were responsible for health improvements. Although the Self-Help Group participants expressed a basic understanding of disease causation leading them to try the new behaviors, it was the improved health of their families that convinced them to continue their new behaviors.

*We now wait to feed the children* [with complementary foods beyond breastmilk] *until they are 6 months old to prevent disease in their lives, but before we fed them at 3 or 4 months and we saw them getting sick easily. Seeing this improvement made it easy to convince other mothers to change their feeding practices.*

-Self-Help Group participant

### Increased social capital

Some Care Group Volunteers expressed that, through their Care Group participation, they built relationships with community leaders and that the health gains realized by the Project increased their standing with those leaders.

*Now, the leaders in the community know us because we formed a committee, and the health of the children has improved.*

*-*Care Group Volunteer

Many Care Group Volunteers spoke of the importance of women forming close relationships with one another in their Self-Help Groups. They cited the importance of group learning as a method for acquiring information and building a sense of community among themselves. They also frequently expressed the value of their new relationships with the community leaders. The Self-Help Group participants stressed the importance of support from community leaders for their own attendance. This support included community leaders reminding them of dates, times, and places of community meetings.

*The community leaders would tell us before, so that we could organize what we had to do and make time for the trainings.*

*-*Self-Help Group participant

Additionally, the Care Group Volunteers expressed that their participation with Care Groups enabled them to establish a reliable communication system by building networks between themselves and Self-Help Group participants as well as between themselves and the Level-1 Promoters. They also spoke of the importance of group learning, which helped them share experiences with each other and led to increased trust among participants. They cited this newfound trust as a fundamental catalyst of their own behavior change. Additionally, Care Group Volunteers and Self-Help Group participants expressed that participation in the Care Groups had increased their participation in other aspects of community life that did not directly relate to health. They stated that taking part in Care Groups allowed them to participate in the community to a greater extent due to increased confidence in their own abilities and because of the heightened respect that community leaders now had for them.

*Currently, we want to go to meetings whether they are about health or not, because they are all important. It is important to have knowledge about the benefits of the activities in our community.*

*-*Care Group Volunteer

## Discussion

Interviews with Care Group participants revealed that Care Group participation was an empowering process. The primary themes that emerged included increased respect accorded to Care Group participants in the community, their greater willingness and ability to make decisions and their greater confidence in those decisions, and the development of stronger bonds among Care Group members as well as with other community members and with community leaders.

Although Care Groups are being implemented widely throughout the world [2] and considerable evidence has accumulated about their effectiveness [15–17, 50], this is the first study that we are aware of that specifically addresses the empowering effects of the Care Group Approach. Care Group participants who participated in our study reported increased self-respect/perceived social status, self-efficacy, decision-making autonomy, and social capital. In their role as health advisors and as leaders of the Care Group process, Level-1 Promoters and Care Group Volunteers reported that they experienced an increase in their social status, at least as they perceived it, as a result of the more favorable view accorded them by the community because of their leadership roles. Self-Help Group participants experienced increased social status due to community perceptions of them as persons with valuable health knowledge. Participating in Care Group activities led to the Care Group Volunteers and Self-Help Group participants being welcomed at other community meetings, which had not been the case previously. Our findings support those of many others who have been able to document improvements in measures of women’s empowerment associated with their participation in other types of self-help/women’s support groups, which﻿ provide an enabling environment for women to gather in small groups regularly for friendships and to address common concerns particularly related to their own health and the health of their children [51–55].

The Self-Help Group participants reported an increased sense of self-worth and self-confidence, which resulted in their greater participation in community affairs. An increased sense of self-efficacy resulted from newly acquired practical knowledge of illness and health gained from their participation in their Self-Help Group as well as from a heightened awareness about the rights of women. Self-efficacy has been shown to affect maternal health service utilization [56], which has implications for the health of mothers and children.

Respondents indicated that they had obtained greater decision-making autonomy in their own homes, especially with respect to husbands and mothers-in-law. Decision-making autonomy was demonstrated through a greater confidence that participants had in their ability to make good decisions. Respondents reported increased decision-making autonomy for both health and non-health decisions, and they stated that their new knowledge translated into increased power over their own lives.

Reports of increased decision-making autonomy among the Care Group participants can be separated into two categories: (1) increased confidence in one’s own decisions (i.e., internal ability to make decisions); and (2) reduction of external barriers to personal decision-making (i.e., less resistance from husbands and mothers-in-law). Thus, respondents became more capable and comfortable making decisions, and they were better able to act upon these decisions. Other studies have shown that increasing the confidence of women in their ability to make decisions can lead to greater decision-making autonomy in the home, and establishing greater equity in household decision-making can improve access to and utilization of maternal and child health services [33–36].

Vital to empowerment is the strengthening of social capital in groups, which affects the allocation of resources and, ultimately, the health of populations [37]. Bonding social capital –the strength of linkages within and among socially homogeneous groups – and bridging social capital –the strength of linkages within and among socially heterogeneous or hierarchal groups – have the potential to improve population health, particularly among the most marginalized members of the population [37, 57, 58]. The meetings every 2 weeks of Care Group Volunteers in their Care Groups and the meetings of the Care Group Volunteers every 2 weeks with their neighbors either individually or through Self-Help Group meetings led to stronger social cohesion (i.e., stronger bonding social capital) than had existed previously as they discussed topics of great importance to all of them. Improvements in bonding social capital may result in greater organization of disempowered groups as well as in a greater capacity to reach a consensus and to move forward with resolve [59]. Improvements in bridging social capital may result in a greater capacity to establish alliances and to obtain leverage with groups having greater power and prestige to affect change [42]. The Care Group process served as a catalyst for building social relationships with other women and with leaders in the community (i.e., bridging social capital).

The Level-1 Promoters and the Self-Help Group participants emphasized the strengthened connections they had with the leaders of the community that came about as a result of the leaders’ logistical and tacit support of their Care Group activities. This produced new forms of bridging social capital that had not previously existed. The Self-Help Group participants and the Care Group Volunteers also spoke about the bonding social capital created from the benefits of group learning and trust built among the participants themselves. Bridging social capital established between the Care Group Volunteers and community leaders was critical for the inclusion of Care Group Volunteers in community meetings. Community leaders alerted Care Group Volunteers about upcoming meeting times and places, which was particularly important because written communications were of no value to the many Care Group Volunteers who were illiterate or semi-literate. On the other hand, Care Group Volunteers and Self-Help Group participants emphasized bonding social capital gained through their Care Group activities as instrumental to giving them the resolve to adopt new recommended behaviors. They cited the importance of group learning as a method for acquiring information and for building a sense of solidarity among themselves, which gave them confidence and support to change their behaviors [60]**.**

Care Group activities relied upon local volunteers who became empowered through their engagement as they came to possess useful knowledge about health and confidence to translate that knowledge into healthy behaviors, leading to visible positive effects on themselves, their children, and on other women and children in the community. As evidenced by the interviews carried out for this study, the Level-1 Promoters, Care Group Volunteers, and Self-Help Group participants each came to take responsibility for the health of their communities, and they acquired the knowledge and agency to translate that knowledge into improved community health.

Additional medium- to long-term follow-up will be necessary to determine whether these female Care Group participants will continue at a more active level of participation in community affairs and whether this will result in more community activities and decisions that benefit women directly. However, the gains in empowerment detected through this study are likely to have contributed to the significant improvements in childhood nutritional status, healthcare utilization, healthy household behaviors, and improvements in mortality demonstrated in the other articles in this series [6–8] as well as to longer-term improvements in health and well-being, as suggested by the literature [61, 62]. The benefits of the Care Group approach for maternal and child health as well as for the social well-being of Care Group participants (in terms of increased self-respect and respect perceived from others in the community, the increased agency, and the stronger social bonds that emerged) provide strong evidence for the value of Care Groups as a valuable component of health programs more broadly. We discuss these and other policy-related themes in the final paper in this series [12].

Care Groups provide a unique opportunity for women to work together, support each other, and learn from each other in an empowering process that enables them to contribute to improvements in the health of themselves, their children, and their neighbors. This provides a unique opportunity for women that lends not﻿ only value and meaning in their own lives, but also to improvements in their perceived social status, self-efficacy, decision-making autonomy, and social capital.

### Limitations

Although the findings of this study provide strong evidence of the empowering effects of the Care Group Approach, some limitations of the study should be mentioned. First, it is possible that the group interview format, used for data collection from Care Group Volunteers and Self-Help Group participants, led to some bias, in that some group members may not have felt comfortable sharing their opinions in a group setting. Individual interviews, as were carried out for the Level-1 Promoters, may have provided more accurate responses but, of course, individual interviews would have required additional time and funding to carry out with the Care Group Volunteers and Self-Help Group participants in order to have the same number of respondents. Second, additional insights might have been gained by interviewing others such as husbands, mothers-in-law, and community leaders. Future research should consider interviewing indirect beneficiaries of the Care Group Approach to understand better the scope of its influence on norms, values, and behaviors. However, these groups were interviewed for the other study on women’s empowerment, which follows this article in our series [10].

Third, since the interviews were not recorded, errors in note-taking and translation may have affected some of the results. The interview responses were translated twice: first from each of the three Maya languages to Spanish in real time during the interview and second from the written Spanish notes to English. The translation of the interview questions from the Maya language to Spanish was made by the researchers as a team to increase fidelity of meaning, but it is possible that some meaning was lost in translation. Fourth, themes were elucidated by an individual researcher, and more themes may have been discovered if the responses had been analyzed by a team of researchers rather than just one individual. However, the final themes were reviewed and approved by the entire team. Finally, we recognize the distinct possibility of social desirability bias—that the respondents were predisposed to provide responses that they thought the interviewers would want to hear [63]. We mitigated this bias by using interviewers who were not otherwise involved in the Care Group activities but were from the local area.

## Conclusion

The Care Group component of the Curamericas Maternal and Child Health Project, 2011–2015, demonstrates strong evidence of women’s empowerment among Level-1 Promoters, Care Group Volunteers, and participants in the Self-Help Groups. They reported that community members respected their new knowledge and capacities, giving them a sense of increased social status among their peers. Their newly acquired knowledge and capacities, giving them enhanced decision-making autonomy at home and greater engagement with the community, reduced their timidity and fear in their interactions with their spouse and mothers-in law and with higher-ranking community members. Finally, the strong relationships that developed within the Care Groups and with neighbors produced stronger bonding social capital, and the collaborations that arose with community leaders and with others in the community through the Care Group process led to new relationships of respect – bridging capital that had not previously been present.

Participation in the Care Group process led to health improvements for themselves and their children that were readily apparent to them, giving them further motivation to participate in Care Group activities, which further enhanced the empowerment process. Participation in Care Groups and Self-Help Groups enabled marginalized indigenous women in rural Guatemala to achieve positive gains in their perceived social status in the community, self-efficacy, decision-making autonomy, and social capital of their networks with multiple benefits for them, their children, their families, and their communities.

## Data Availability

All of the Project reports, de-identified data, as well as publications about the Expanded CBIO+ Approach cited in this article are available from the corresponding author on request.

## Appendix

### Interviewer Guide Questions

**For the Community Facilitators (CFs)**

1. Tell me what you do as an CF.
  - What do you like about it?
  - What don’t you like about it?
  - What would make your work as a CF easier and more effective?
2. How effective do you think you were training the *Comunicadoras* to teach life-saving knowledge and to get women in the Self-Help Groups (SHGs) to change their behavior?
  - What helped you be an effective teacher?
  - What made the teaching difficult?
  - How could you have taught them better?
3. How well did the Educators train you to record and communicate vital events?
  - What facilitated your ability to record and communicate vital events?
  - What made it difficult?
  - What could have made it easier to record and communicate the vital events?
4. How effective do you think the Educator was at teaching you life-saving knowledge and at teaching you to teach the *Comunicadoras*?
  - What helped her be an effective teacher?
  - What made the teaching difficult?
  - How could she have taught you better?
5. How effective were the training materials you were provided?
  - How could the training materials be improved?
6. What behavior changes have you noticed in the *Comunicadoras*?
  - What behavior changes that you wanted to see did not happen?
  - What do you think helped the *Comunicadoras* change their behavior?
  - What do you think prevented the *Comunicadoras* from changing their behavior?
7. Do you feel that you have more or less control over your life now compared to before you were a CF? Please elaborate.
  - Do you have more or less control over your own decisions now?
  - Has your status in the community changed at all?
  - Do you think that you could participate more easily in community meetings and projects? Explain.
8. What do you think made it easy for the *Comunicadoras* to keep attending and participating in the Care Groups?
  - What do you think made it difficult for them?
  - What improvements could be made to keep the *Comunicadoras* more interested and engaged?
9. What recommendations do you have for improving the Care Groups and the SHGs?

**For the Comunicadoras**

1. Tell me what you do as a *Comunicadora*
  - What do you like about it?
  - What don’t you like about it?
  - What would make your work as a *Comunicadora* easier and more effective?
2. How effective do you think you were in training the SHGs to teach life-saving knowledge and to change their behavior?
  - What helped you be an effective teacher?
  - What made the teaching difficult?
  - How could you have taught them better?
3. How well did the Community Facilitators train you to record and communicate vital events?
  - What facilitated your ability to record and communicate vital events?
  - What made it difficult?
  - What could have made it easier to record and communicate the vital events?
4. How effective do you think the CF was at teaching you life-saving knowledge and at teaching you to teach the SHGs?
  - What helped her be an effective teacher?
  - What made the teaching difficult?
  - How could she have taught you better?
5. How effective were the training materials you were provided?
  - How could the training materials be improved?
6. What behavior changes have you noticed in the SHG women?
  - What behavior changes you wanted to see did not happen?
  - What do you think helped the SHG women change their behavior?
  - What do you think prevented the SHG women from changing their behavior?
7. Do you feel that you have more or less control over your life now compared to before you were a *Comunicadora*? Please elaborate.
  - Do you have more or less control over your own decisions now?
  - Has your status in the community changed at all?
  - Do you think that you could participate more or less easily in community meetings and projects? Explain.
8. What do you think made it easy for the SHG women to keep attending and participating in the Self-Help Groups?
  - What do you think made it difficult for them?
  - What improvements could be made to keep the SHG women more interested and engaged?
9. What recommendations do you have for improving the Care Groups and the SHGs?

**For the Self-Help Groups (SHGs)**

1. Tell me what you do in the SHG.
  - What do you like about the SHG?
  - What don’t you like about it?
2. How effective do you think the *Comunicadora* was at teaching you life-saving knowledge?
  - What helped her be an effective teacher?
  - What made the teaching difficult?
  - How could she have taught you better?
3. How effective were the training materials used?
  - How could the training materials be improved?
4. What behavior changes have you noticed in yourself and each other?
  - What helped you change your behavior?
  - What made it difficult?
5. Do you feel that you have more or less control over your life now compared to before you participated in the SHG? Please elaborate.
  - Do you have more or less control over your own decisions now?
  - Has your status in the community changed at all?
  - Do you think that you could participate more or less easily in community meetings and projects? Explain.
6. What things made it easy for you to come to and participate in the SHGs?
  - What things made it difficult to come to and participate in the SHGs?
7. What recommendations do you have to improve the SHGs?

## Abbreviations

CBIO: Census- based, Impacted-oriented Approach
CBIO+: Expanded CBIO Approach

## Footnotes

1 Municipalities in Guatemala are the equivalent of districts or counties in many countries – namely geographically contiguous areas as opposed to the use of this term in many countries to refer to non-contiguous towns and cities.

2 Many of the communities in the Program area were difficult to reach because of the terrain and lack of roads. Given the budget and time constraints of the evaluation, we could not include the more isolated communities in this aspect of the evaluation.
